# Comparison of visual evoked potential variability in eyes affected by optic neuritis and fellow eyes

**DOI:** 10.1007/s10633-025-10061-y

**Published:** 2025-11-10

**Authors:** Marie Chutná, Jan Kremláček, Miroslav Kuba, Zuzana Kubová, Jana Szanyi, František Vít, Jana Langrová

**Affiliations:** 1 Department of Pathological Physiology, Faculty of Medicine in Hradec Kralove, Charles University, Simkova 870, 500 03 Hradec Kralove, Czech Republic; 2 Department of Medical Biophysics, Faculty of Medicine in Hradec Kralove, Charles University, Simkova 870, 500 03 Hradec Kralove, Czech Republic

**Keywords:** Signal‒to‒noise ratio, Averaging methods, Latency jitter, Trial–to–trial variability

## Abstract

**Purpose:**

This study compared the variability of visual evoked potential (VEP) in response to stimulation of eyes affected by unilateral optic neuritis with that of fellow (non-affected) eyes.

**Methods:**

Pattern-reversal VEP (PVEP) and motion-onset VEP (MVEP) recordings from thirty-six subjects with unilateral optic neuritis at different intervals from disease onset were retrospectively evaluated, and differences in the following parameters were compared: signal‒to‒noise ratio (SRN), interquartile range of the response jitter (jitter IQR), and number of trials corresponding to the average response (corresponding N).

**Results:**

In the PVEP recordings, the P1 peak times of the fellow eyes were significantly shorter than those of the affected eyes (Cohen’s d = -1.470, p < 0.001). P1 amplitudes were significantly greater in fellow eyes (*d* = 1.17, *p* < 0.001). Significant differences were found in the SNR (*d* = 0.782, *p* < 0.001), jitter IQR (*d* = -0.874, ***p*** < 0.001), and corresponding N (*d* = 0.700, *p *< 0.001).

MVEP presented significantly shorter N2 peak times in fellow eyes than in affected eyes (*d* = 0.840, *p* < 0.01) and significantly greater amplitudes (*d* = 0.494, *p* = 0.002). There was a significant difference in the SNRs (*d* = 0.440, *p* = 0.01) and corresponding N values (*d* = 0.415, *p* = 0.01). There was no difference in the jitter IQR (*d* = 0.143, *p* = 0.230).

**Conclusions:**

The increased variability in eyes affected by optic neuritis compared with fellow eyes (in particular, in pattern-reversal VEP, which predominantly represents the activity of the macular–papillary fibers of the optic nerves) may represent important pathophysiologic features and may add valuable information to diagnostics via VEP examinations.

## Introduction

The visual evoked potential (VEP) provides objective functional assessment of the visual pathway, and since its first clinical usage [1], it has proven to be a useful tool for both clinical and experimental investigations of visual pathways [2].

Averaging is often used for VEP processing. This method has enabled tremendous progress in VEP investigations and has made this method available for routine clinical diagnostics [3]. However, evoked responses of the central nervous system are rather variable even for a precisely defined stimulus, which is kept constant for all trials; thus, due to averaging, some information about the variability of VEP could be lost [4]. The degree of variability could be affected by several factors, e.g., the current conditions of the examined persons, their ability to maintain attention during the examination, by brain adaptation to the visual stimuli [5–7], and, as several studies have suggested, the level of variability in the CNS may be functionally relevant, and may even be linked to some pathological conditions [8–13]. Increased variability may also affect the final trace gained by the averaging of single trials and possibly interfere with test interpretation. For example, time fluctuation of single trials, a phenomenon called latency jitter, may cause an amplitude decrease in averaged recordings or can affect the peak shape, so assessing a VEP on the basis of only the nominal peak time may not be sufficient in some cases [9, 14–16]. Therefore, it would be useful to explore whether there is increased variability in diseases investigated by VEP, and whether the assessment of VEP response variability might serve as beneficial additional information to increase the diagnostic yield of the investigation in general. Assessment of variability is often based on single trial examinations, but our aim was to find simple and easily evaluable parameters that reflect the variability of VEP and to examine whether the variability is greater in a pathological condition. For this purpose, we evaluated variability in eyes affected by unilateral optic neuritis compared with fellow eyes.

Optic neuritis (ON) is an inflammatory disorder of the optic nerve that causes unilateral acute impairment of vision. It can affect one or both eyes either simultaneously or successively [17]. There could be several underlying pathologies, but one of the most common is multiple sclerosis [18]. The diagnosis of ON was chosen because it represents one of the most common and thoroughly studied pathologies for VEP examination, and when it is unilateral, it enables variability of affected and healthy eyes to be compared within the same subject to the other eye, thus minimizing the interference from inter-subject variability which is quite high [15].

Two types of VEP stimuli were analyzed in this study: pattern-reversal VEP (PVEP), which represents the most frequently employed VEP, and motion-onset VEP (MVEP). MVEP are routinely examined in our laboratory because a wider range of stimulus types can increase the diagnostic sensitivity of VEP examination [2, 19], and in some cases, especially if the magnocellular pathway is predominantly affected, pathological findings can be observed only in MVEP, whereas PVEP is normal [20–22].

## Subjects and methods

### Subjects

The data represent retrospective VEP analysis of patients saved at the database of the Electrophysiological Laboratory in the Department of Pathological Physiology in Hradec Kralove from 2010 to 2024 (examined under strictly uniform conditions).

Thirty-six subjects were chosen for the assessment, 13 males and 23 females, aged 22–50 years, with a mean age of 33.9 years (see Table 1 for demographic data). There were more females in the sample, which is consistent with the incidence of optic neuritis being greater in females [23].

**Table 1:** Demographics and visual acuity at the time of testing

Patient no	Age	Gender	VA FE	VA AE	logMAR VA FE	logMAR VA AE
1	42	F	1.00	0.25	0.00	0.60
2	24	M	1.00	0.13	0.00	0.90
3	46	M	0.80	0.20	0.10	0.70
4	50	F	0.25	0.13	0.60	0.90
5	22	F	0.40	0.10	0.40	1.00
6	38	F	0.80	0.25	0.10	0.60
7	30	M	1.00	1.00	0.00	0.00
8	24	F	1.00	0.40	0.00	0.40
9	38	M	0.80	0.50	0.10	0.30
10	22	F	0.67	0.17	0.17	0.77
11	36	F	1.00	0.50	0.00	0.30
12	26	F	1.00	0.67	0.00	0.17
13	46	M	0.80	0.80	0.10	0.10
14	25	F	0.50	0.13	0.30	0.90
15	35	M	1.00	0.10	0.00	1.00
16	34	M	1.00	0.80	0.00	0.10
17	45	F	1.00	0.10	0.00	1.00
18	39	F	1.00	0.20	0.00	0.70
19	46	M	1.00	0.33	0.00	0.48
20	47	F	0.80	0.20	0.10	0.70
21	31	F	0.50	0.25	0.30	0.60
22	33	F	0.40	0.20	0.40	0.70
23	31	M	0.25	0.10	0.60	1.00
24	41	M	0.80	0.13	0.10	0.90
25	23	F	1.00	1.00	0.00	0.00
26	22	F	1.00	0.10	0.00	1.00
27	31	F	0.67	0.67	0.17	0.17
28	36	F	1.00	0.33	0.00	0.48
29	28	M	1.00	0.80	0.00	0.10
30	39	M	0.25	0.17	0.60	0.77
31	26	F	1.00	0.50	0.00	0.30
32	44	F	1.00	1.00	0.00	0.00
33	23	M	0.40	0.25	0.40	0.60
34	28	F	1.33	0.80	-0.12	0.10
35	32	F	1.00	1.00	0.00	0.00
36	36	F	0.80	0.40	0.10	0.40

VA – Visual acuity; FE – Fellow eye; AE – affected eye

The inclusion criteria were as follows: age 18–65 years, visual acuity assessment available, readable VEP in both eyes for at least one of the stimuli: pattern-reversal VEP (PVEP)/motion-onset VEP (MVEP), and clinical diagnosis of unilateral optic neuritis based on suggestive symptoms (confirmed by the clinical expert who made the referral for the VEP investigation) and supported by a paraclinical test, an abnormal VEP. The typical clinical course and symptoms, together with supporting evidence from one paraclinical test, are regarded as sufficient for the diagnosis of optic neuritis [24, 25]. To confirm an abnormal VEP, we assessed the peak time (latency) of the main peak P1/N2, its amplitude and the interocular difference. The assessment was based on comparisons with normative data for the adequate age group set in our laboratory. Optic nerve affection was defined as a prolonged nominal peak time and/or significant interocular nominal peak time/amplitude asymmetry or the absence of the P1/N2 wave.

The exclusion criteria included a history of optic neuritis in the other eye, other ocular comorbidities and diseases potentially affecting vision (such as diabetes mellitus, amblyopia, and neuroborreliosis). Patients older than 65 years were not included because it cannot be ruled out that there may be an effect of even healthy aging on VEP variability [26]. Patients younger than 18 years were not included because the visual system is known to mature during childhood and adolescence, which is reflected especially by the response to motion stimulation [27].

### Ethics

All the patients clearly agreed with the testing and possible subsequent use of their data for research. This analysis was a part of a larger research project that adhered to the tenets of the Declaration of Helsinki and was approved by the Ethical Committee of the Medical Faculty in Hradec Kralove.

### Visual acuity

Information on visual acuity was obtained via the Landolt C test, and the logMAR score was used for statistical analysis (see Table 1).

### Visual stimulation

Two types of stimulation were chosen for the analysis: pattern reversal VEP (PVEP) and motion onset VEP – centrifugal/centripetal motion (‘‘expansion/contraction motion” – MVEP).

There are some differences in our parameters from the current ISCEV standards for PVEP examination [28]. The reason for this is that our laboratory would like to maintain the continuity of our stimulus and recording parameters and published results for a long period of time, starting in approximately 1990.

Pattern-reversal stimulation (PVEP) was elicited using a black and white checkerboard (contrast 96% according to Michelson) with check sizes of 40’ reversing at a frequency of 2/s. For the standard PVEP examination we routinely use three checker sizes (40´, 20´and 10´ or more in the case of low visual acuity). Smaller check sizes were not analyzed because in many patients, the responses to smaller checks were not well readable and therefore not suitable for this type of analysis.

Motion onset VEP (MVEP) was elicited using the radial motion of low luminance contrast (10%) circles with randomly selected centrifugal or centripetal (‘‘expansion/contraction ”) directions [20]. The spatial frequency of the structure decreased, and the motion velocity increased from the center (fixation point) toward the periphery, respecting the size of the retinal receptive fields and the sensitivity to motion velocity across the retina [29]. The temporal frequency of this stimulus was 5 Hz in all parts of the visual field. The timing was 200 ms of motion followed by a one second interstimulus interval, during which the stationary pattern was presented, to prevent adaptation to motion.

All visual stimuli were generated via VSG 2.5; CRS Ltd., UK on a 21’’ Iyama monitor (Japan) with a vertical frequency of 105 Hz. The stimulus field subtended 37 × 28° at a viewing distance of 0.6 m, and the average luminance of 17 cd/m^2^ was constant. We use rather low luminance, different from ISCEV guidelines [28], because it may lead to increased examination sensitivity in optic nerve pathologies without increasing falsely positive results [19, 30].

The electrophysiological examination was performed in a Faraday cage to decrease the electromagnetic noise. Patients were instructed to keep their gaze on the red cross in the center of the screen during recording. To minimize the influence of poor fixation, we used an infrared CCD camera to check the correct fixation of the stimulus field center, and according to an assessment of examinators, all the chosen patients fixated quite well. All recordings were performed monocularly with the non-tested eye covered with an eye patch. All patients were tested with their best spectacle correction if needed, and no additional short-distance correction (for 0.6 m viewing distance) was provided.

Ag–AgCl electrodes for the VEP recording were placed according to the International 10–10 system [31] using pseudounipolar derivations from the midline Oz, Pz, Cz and Fz. Two additional electrodes were placed 5 cm to the left (O_L_) and right (O_R_) of the Oz position as originally suggested by Göpfert et al. [32], since at these locations, MVEP has a maximum amplitude (dependent on individual lateralization). The reference electrode was placed on the right ear lobe.

EEG poststimulus epochs of 440 ms duration were sampled at 500 Hz. The signal was amplified 20,000 times (Contact Precision Instruments – PSYLAB, System 5, UK) and filtered to include frequencies ranging from 0.3 to 45 Hz. Forty single trials were recorded and averaged, because this number of trials in our recording settings provides a good signal‒to‒noise ratio.

We standardly use more (six) different types of stimulation for routine clinical VEP investigations (40´, 20´and 10´ sizes of PVEP and three types of MVEP) because we believe it increases the diagnostic yield of the investigation [2]. However, the examination takes longer, and patients’ fatigue and decreased attention might interfere with the results [33]. For these reasons, we record forty single responses, not the hundred responses recommended by ISCEV [28].

### Variability analysis

To assess VEP variability, we set two parameters that reflect the variability of the recordings:

1. *jitter IQR* represents the interquartile range of the latency shifts (jitters) in milliseconds applied to individual responses (epochs) during the alignment process with a template waveform. Single responses were subsequently shifted to match an iteratively constructed average potential (template) using Woody's approach [34]. This iterative process continued until the root mean square (RMS) of the difference between succeeding averages was less than 0.1, indicating convergence and optimal alignment. The fitting between each response and the template was based on the cross-correlation function computed over a 1–440 ms time window, encompassing the entire recorded epoch of the VEP response.

The latency shifts applied to each epoch were recorded as jitters, reflecting the adjustments needed for optimal alignment with the template. We evaluated the variability in response timing across trials by calculating these jitters' interquartile range (IQR). A smaller jitter IQR indicates more consistent neural response timing across trials, suggesting lower variability in neural processing. Conversely, a larger IQR signifies greater variability in the timing of VEP responses.

2. *corresponding N* represents the number of epochs that closely correspond with the template VEP within a ± 30 ms jitter window. By limiting the jitter window, we avoid falsely enhancing noise due to misalignment with background EEG activity. The ± 30 ms was selected to be shorter than a shift that erroneously aligned counter-phase alpha activity to the average.

In addition, we analyzed the *signal-to-noise ratio* (SNR) because it is an important parameter that reflects the actual change in the response. If we assume constant noise levels, a decrease in the SNR may reflect both a diminished signal amplitude and increased time variability. The SNR calculation was based on the ratio between the root mean square (RMS) of the average evoked response and the reference noise calculated using the "plus-minus" principle [35].

The average waveform is computed from single trials to estimate the signal level. The plus-minus averaging technique is employed to assess the noise level. This involves inverting the polarity of half of the recorded trials before averaging. When these inverted and non-inverted averages are combined, the consistent VEP signals cancel each other out because of their opposite polarities, effectively isolating the random noise components.

The RMS values of the signal and noise waveforms are then calculated. The SNR is then determined by dividing the RMS of the averaged VEP response by the RMS of the noise estimated through the plus-minus method

$${\mathrm{SNR}} = \frac{{\text{RMS of Average Response}}}{{\text{RMS of Reference Noise}}}$$

### Analysis of the recordings

Recordings from the Oz derivation were evaluated in all patients for PVEP stimulation. For MVEP, the optimal derivation, which represents the derivation with the largest amplitude of the main motion-specific peak N2 (with a normal peak time of app. 160–200 ms), [20] was chosen for each patient individually.

The recordings were evaluated in MATLAB R2023a (MathWorks, USA).

In each recording, the peak time and the interpeak amplitude of the main peak were determined, as were the signal‒to‒noise ratio (SNR), the jitter IQR, and the corresponding N (for definitions, see above).

In three participants the PVEP was undetectable; in three other participants, the MVEP was undetectable; thus, thirty-three PVEP and thirty-three MVEP recordings were analyzed.

### Statistical analysis

Statistical tests were performed and graphs were generated with Jamovi (version 2.3.17) [36].

Based on the Shapiro–Wilk W test, some of the data sets did not fulfill the criteria of normality; therefore, they were summarized with the median and ranges, and the Wilcoxon pair test was used for the assessment of the differences in variability between affected and fellow eyes. Cohen’s d was also calculated to illustrate the effect size corresponding to the parametric tests.

The Spearman correlation coefficient was used to test the correlation between amplitude magnitude and variability across trials (reflected by the SNR, jitter IQR and corresponding *N*). Within subject (paired) comparisons were chosen to eliminate interindividual variability in VEP parameters.

Significance was assessed at the alpha level 0.05. For multiple testing correction, we used the Holm‒Bonferroni correction to adjust the alpha level appropriately [37].

Clustered ROC (Receiver Operating Characteristic) analysis was performed to illustrate the discriminative performance of the following parameters. ROC analysis was conducted in the *R* environment (ver. 4.3.1) [38] based on a nonparametric analysis of clustered ROC [39, 40]

## Results

**Fig. 1:**
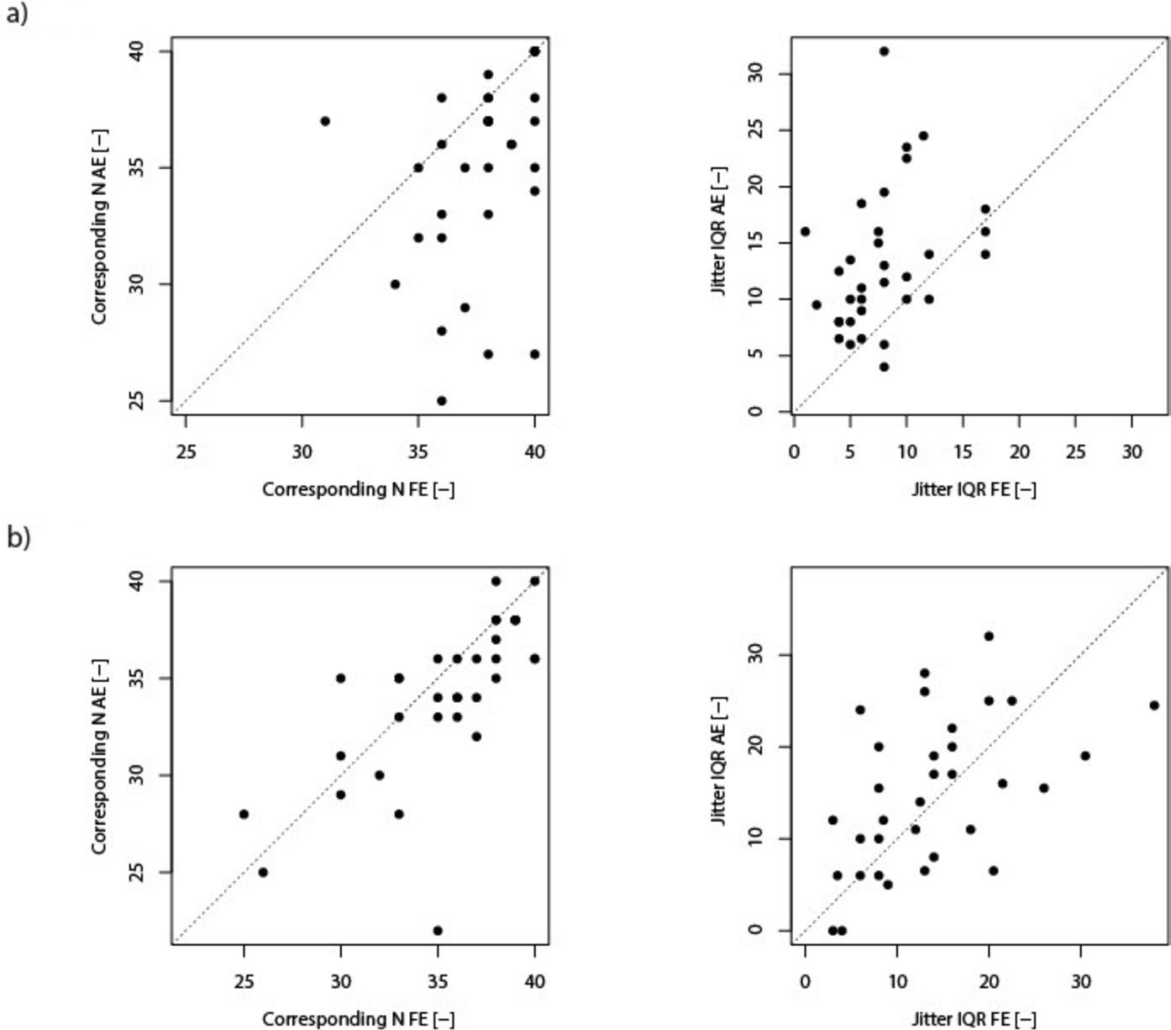
Pattern-reversal VEP (PVEP) (1a) and motion-onset VEP (MVEP) (1b) comparisons of jitter IQR and Corresponding *N* values between fellow and affected eyes.Scatter plots illustrating the relationship between variability parameters values for each patient, comparing the fellow eye (FE, horizontal axis) to the affected eye (AE, vertical axis). Each dot represents an individual patient’s data point. The dotted diagonal line indicates the identity line (i.e., where fellow and affected eye values would be equal). *Panel 1b: MVEP – Left Plot* Scatter plot depicting *corresponding N* values. More points falling below the identity line suggest that the fellow eye often has higher corresponding N values than the affected eye (*p* = 0.01). Right Plot: Scatter plot depicting *jitter IQR* values of the fellow and affected eye. Here, the difference was not statistically significant (*p* = 0.23).

**Fig. 2:**
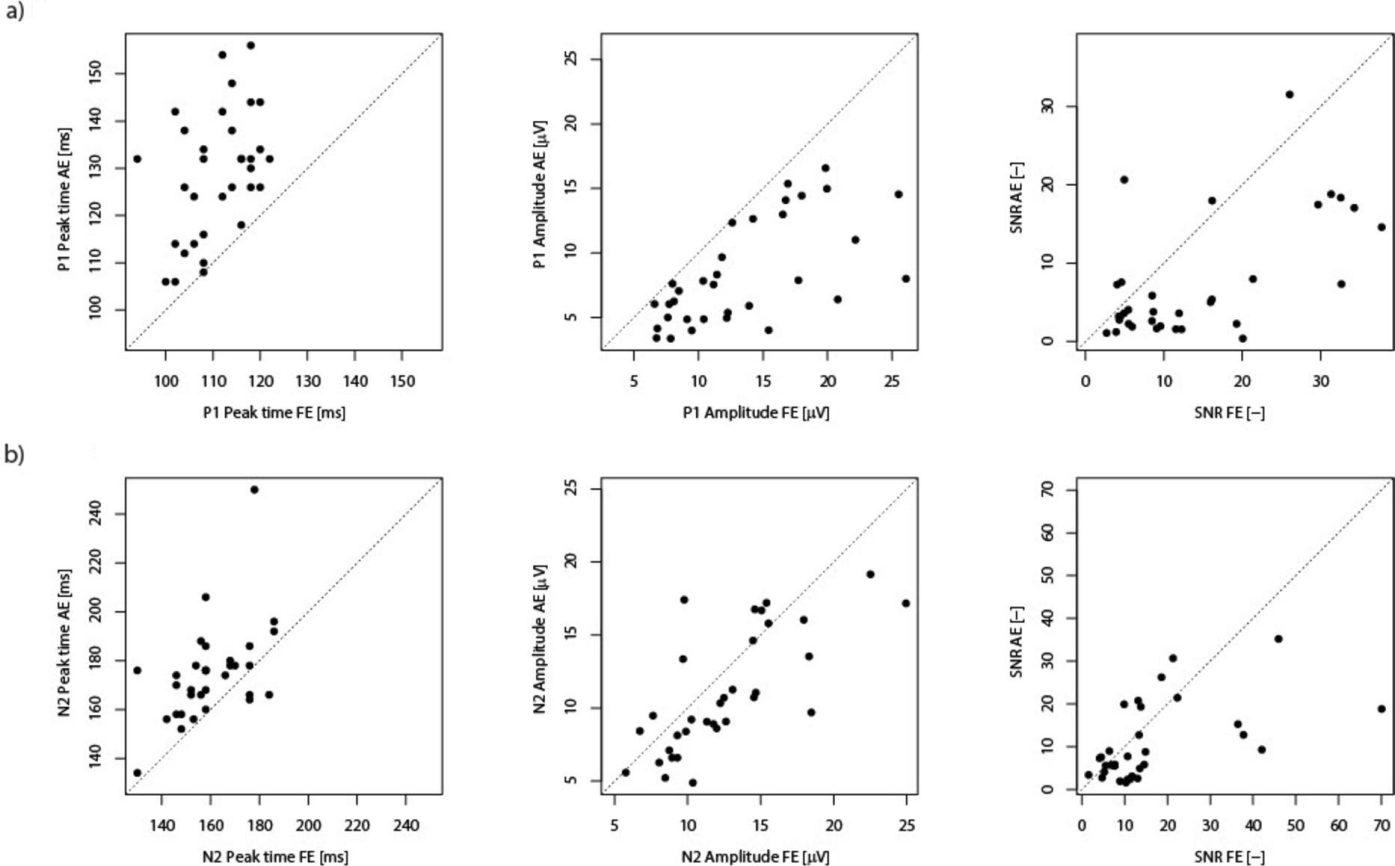
Pattern-reversal VEP (PVEP) (2a) and motion-onset VEP (MVEP) (2b) comparisons of the nominal peak time, amplitudes and SNR between fellow and affected eyes. Scatter plots illustrating the relationship between nominal peak time, amplitudes and SNR values for each patient, comparing the fellow eye (FE, horizontal axis) to the affected eye (AE, vertical axis). Each dot represents an individual patient’s data point. The dotted diagonal line indicates the identity line (i.e., where fellow and affected eye values would be equal). *Panel 1a: PVEP—Left Plot* Scatter plot illustrating *P1 peak times.* A clear asymmetry around this line, with almost all points falling above it, shows that the affected eye has longer P1 peak time than the fellow eye (*p* < 0.001). Middle Plot: Scatter plot depicting *P1 amplitudes.* A clear asymmetry around the identity line, with almost all points falling below it, shows that the affected eye has lower P1 amplitude than the fellow eye (*p* < 0.001). Right Plot: Scatter plot illustrating *SNR* values with more points falling below the identity line suggesting that the fellow eye often has higher SNR values than the affected eye (*p* < 0.001). *Panel 1b: MVEP – Left Plot* Scatter plot depicting *N2 peak times.* A visible asymmetry around the identity line, with more points falling above it, shows that the affected eye has longer N2 peak time than the fellow eye (*p* < 0.001). Middle Plot: Scatter plot illustrating the relationship between *N2 amplitudes* with more points falling below the identity line demonstrating greater amplitude of fellow eye compared to the affected eye (*p* = 0.002). Right Plot: Scatter plot illustrating *SNR* values with more points falling below the identity line suggesting that the fellow eye often has higher SNR values than the affected eye (*p* = 0.01).

**Fig. 3:**
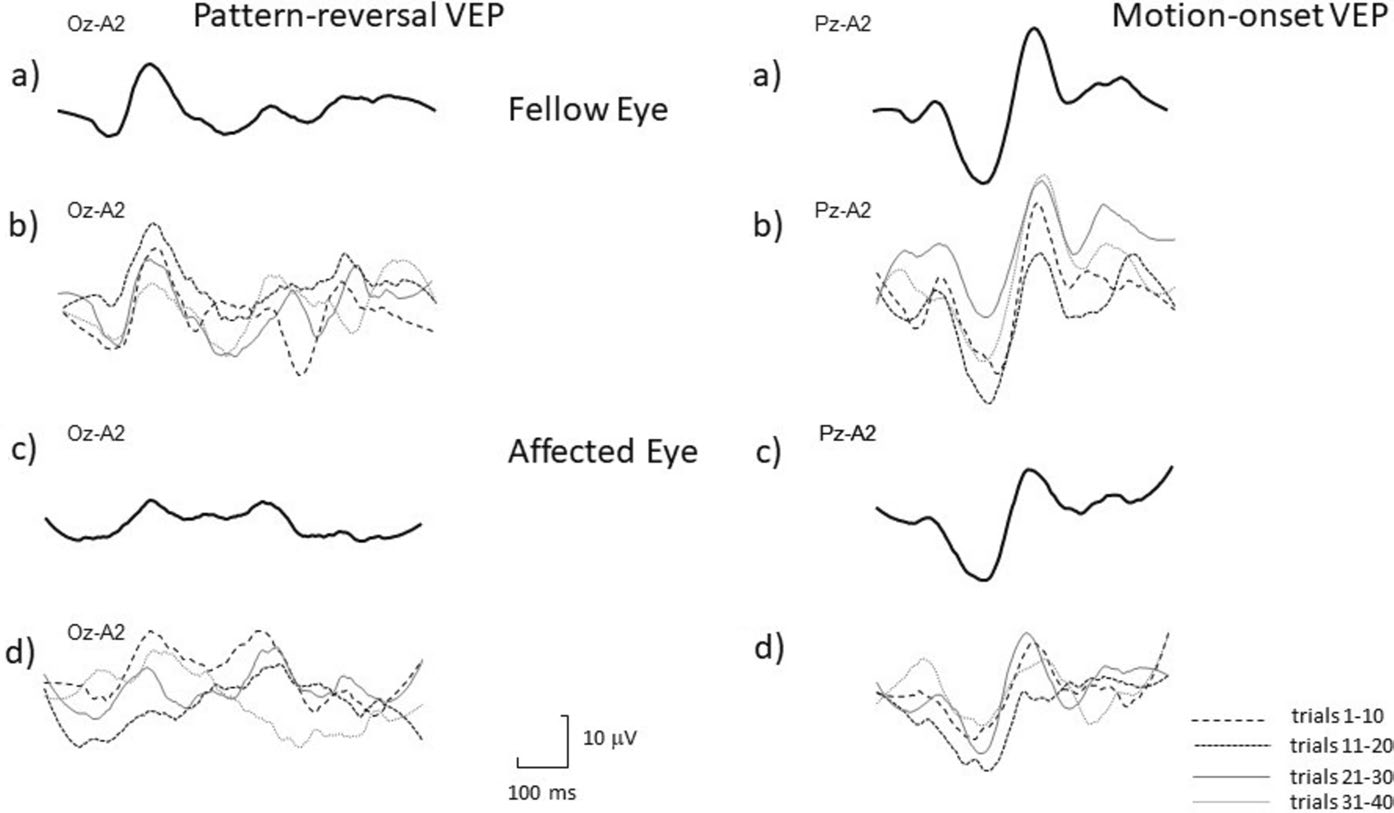
Illustrative traces of response variability in a patient with unilateral optic neuritis (female, age 22). 1a) PVEP fellow eye – final trace, 1b) PVEP fellow eye – final trace divided into four subgroups of trials (quarters), 1c) PVEP affected eye – final trace, 1d) PVEP affected eye – final trace divided into four subgroups of trials, 2a) MVEP fellow eye – final trace, 2b) MVEP fellow eye – final trace divided into four subgroups of trials, 2c) MVEP affected eye – final trace, 2d) MVEP affected eye – final trace divided into four subgroups of trials

The results for pattern-reversal VEP (PVEP) and motion-onset VEP (MVEP) are summarized in Tables 2 and 3 and are plotted in Figs. 1, 2, and 3

**Table 2:** Summarized results of the Pattern reversal VEP (PVEP) parameters.

	FE Median (Q1, Q3)	AE Median (Q1, Q3)	FE – AE (95% CI)	Wilcoxon paired test: p value
P1 peak time [ms]	112.0 (106.0, 118.0)	132.0 (118.0, 138.0)	(-inf, -14.0)	*p* < 0.001
P1 amplitude [µV]	12.2 (8.5, 16.9)	7.6 (5.0, 12.3)	(3.1, inf)	*p* < 0.001
SNR [-]	9.6 (4.9, 20.1)	3.8 (2.3, 8.0)	(4.06, inf)	*p* < 0.001
Jitter IQR [ms]	7.5 (5.0, 10.0)	12.0 (9.0, 16.0)	(-inf, -3.25)	*p* < 0.001
Corresponding N [-]	38.0 (36.0, 40.0)	36.0 (33.0, 38.0)	(2.0, inf)	*p* < 0.001

The last column includes p-values for each paired test, which indicate the significance of the observed differences between AE and FE. Additionally, 95% confidence intervals (CI) for the estimated difference in means between the eyes are provided, showing the range within which the true difference is likely to fall with 95% confidence. Values (Q1, Q3) represent lower and upper quartiles. Statistically significant differences that were subjected to Holm-Bonferroni correction are marked in bold, appropriate alpha levels were as follow: 0.01, 0.0125, 0.0166, 0.025, and 0.05 FE – fellow eye; AF – affected eye; SNR – signal-to-noise ratio; CI – Confidence Interval

*Panel 1a: PVEP—Left Plot* Scatter plot depicting *corresponding N* values. A visible asymmetry around the identity line, with most points falling below it, suggests that the fellow eye often has higher corresponding N values than the affected eye (*p* < 0.001). Right Plot: Scatter plot depicting *jitter IQR* values*.* A visible asymmetry around the identity line, with most points falling above it, suggests that fellow eye often has lower *jitter IQR* values than the affected eye (*p* < 0.001), indicating more consistent temporal response patterns in the fellow eye.

**Table 3:** Summarized results of the Motion-onset VEP (MVEP) parameters.

	FE Median (Q1, Q3)	AE Median (Q1, Q3)	FE – AE (95% CI)	Wilcoxon paired test: p value
N2 peak time [ms]	158.0 (152.0, 170.0)	174.0 (166.0, 178.0)	(-inf, -9.00)	p < 0.001
N2 amplitude [µV]	12.0 (9.3, 14.7)	9.7 (8.4, 14.6)	(0.552, 0.780)	p = 0.002
SNR [-]	11.3 (7.5, 14.9)	7.3 (4.1, 15.3)	(1.01, inf)	p = 0.01
Jitter IQR [ms]	13.0 (8.0, 18.0)	15.5 (8.0, 20.0)	(-inf, 1.25)	p = 0.23
Corresponding N [-]	36.0 (33.0, 38.0)	35.0 (33.0, 36.0)	(0.5, inf)	p = 0.01

The last column includes *p*-values for each paired test, which indicate the significance of the observed differences between AE and FE. Additionally, 95% confidence intervals (CI) for the estimated difference in means between the eyes are provided, showing the range within which the true difference is likely to fall with 95% confidence. Values (Q1, Q3) represent lower and upper quartiles. Statistically significant differences that were subjected to Holm-Bonferroni correction are marked in bold, appropriate alpha levels were as follow: 0.01, 0.0125, 0.0166, 0.025, and 0.05 FE – fellow eye; AF – affected eye; SNR – signal-to-noise ratio; CI – Confidence Interval

### Pattern-reversal VEP (PVEP)

Significant differences between fellow and affected eyes were found in the P1 peak time, P1 amplitude, signal–to–noise ratio (SNR), jitter IQR and corresponding N.

The median P1 peak time was 112.0 ms (Q1, Q3: 106.0 ms, 118 ms) for the fellow eyes and 132.0 (118.0, 138.0) for the affected eyes. The P1 peak times in the fellow eyes were significantly shorter than those in the affected eyes (*d* = -1.470, *p* < 0.001; 95% Confidence Interval: -inf, -14.0) as expected, because the peak time was used as a diagnostic marker and selection criterion.

The P1 interpeak amplitudes of the fellow eyes, with a median of 12.2 µV (Q1, Q3: 8.5 µV, 16.9 µV), were significantly greater (*d* = 1.170, *p *< 0.001, 95% CI: 3.1, inf) than those of the affected eyes, with a median of 7.6 µV (5.0, 12.3 µV).

The median SNR for the fellow eyes was 9.6 (Q1, Q3: 4.9, 20.1), which was significantly higher than that for the affected eyes (*d* = 0.782, *p* =  < 0.001(95% CI: 4.06, inf), where the median was 3.8 (Q1, Q3: 2.3, 8.0).

The median jitter IQR was 7.5 ms (Q1, Q3: 5.0, 10.0 ms) for the fellow eyes and 12.0 ms (Q1, Q3: 9.0, 16.0 ms) for the affected eyes, which represent a significant difference, *d* = -0.874, *p *< 0.001 (95% CI: -inf, -3.25).

The values of the corresponding N were also significantly different, *d* = 0.700, *p* < 0.001 (95% CI: 2.0, inf). The median values for the fellow eyes were 38.0 (36.0, 40.0) and for the affected eyes 36.0 (33.0, 38.0).

Spearman correlation coefficients were calculated to confirm the assumption that increased variability may underlie a reduction in amplitude. The Spearman correlation coefficient between the amplitudes and SNRs was 0.558 (*p* < 0.001) for the affected eyes and 0.592 (*p* < 0.001) for the fellow eyes. The Spearman correlation coefficient between the amplitudes and corresponding N was 0.631 (*p* < 0.001) for the affected eyes and 0.351 (*p* = 0.022) for the fellow eyes. The Spearman correlation coefficient between the amplitudes and jitter IQRs was -0.514 (*p* = 0.001) for the affected eyes and -0.546 (*p* < 0.001).

ROC analysis for illustration of the discrimination power of the assessed parameters revealed the highest area under the curve (AUC) of 86.5% (95% confidence interval: 79.44%-93.56%) for P1 peak time, for the SNR (74.75; 65.86–83.64%), for the jitter IQR (78.01; 66.24–86.77%), and for the corresponding N (71.58; 62.24–86.77%) the AUC was smaller, although not significantly.

### Motion-onset VEP (MVEP)

Significant differences between fellow and affected eyes were found in the peak times of the main motion-onset specific N2 peaks and their amplitudes and in the SNRs and corresponding N values of the MVEP recordings. No significant difference was found in the jitter IQR.

The peak times of the main motion-onset specific N2 peaks were significantly shorter (*d* = -0.840, *p* < 0.001 95% CI: -inf, -9.00) in the fellow eyes, with a median of 158.0 ms (Q1, Q3: 152.0, 170.0 ms) than in the affected eyes, with a median of 174.0 ms (166.0 ms, 178.0 ms). The amplitudes were greater (*d* = 0.494, *p* = 0.002).

The amplitudes of N2 peak were significantly greater (d = 0.494, p = 0.002; 95% CI: 0.552, 0.780) in the fellow eyes, median 12.0 µV (Q1, Q3: 9.3 µV, 14.7 µV), than in the affected eyes, with a median of 9.7 µV (Q1, Q3: 8.4 µV, 14.6 µV).

The median SNR for the fellow eyes was 11.3 (Q1, Q3: 7.5, 14.9), which was significantly higher than that for the affected eyes (*d* = 0.440, *p *= 0.010; 95% CI: 1.01, inf), where the median was 7.3 (Q1, Q3: 4.1, 15.3).

No significant difference was found in the jitter IQR (*d* = -0.143, *p* = 0.230; 95% CI: -inf, 1.25), where the median was 13.0 ms (Q1, Q3: 8.0, 18.0 ms) for the fellow eyes and 15.5 ms (Q1, Q3: 8.0, 20.0 ms) for the affected eyes.

The values of the corresponding N differed significantly, *d* = 0.415, *p* = 0.010; 95% CI: 0.5, inf). The median values for the fellow eyes were 36.0 (Q1, Q3: 33.0, 38.0) and for the affected eyes 35.0 (Q1, Q3: 33.0, 36.0).

Spearman correlation coefficients were also calculated for the MVEP recordings to confirm the assumption that increased variability may underlie a reduction in amplitude: The Spearman correlation coefficient between the amplitudes and SNRs was 0.380 (*p* = 0.015) for the affected eyes and 0.364 (*p* = 0.019) for the fellow eyes. The Spearman correlation coefficient between the amplitudes and corresponding N was 0.568 (*p* < 0.001) for the affected eyes and 0.573 (*p* < 0.001) for the fellow eyes. The Spearman correlation coefficient between the amplitudes and jitter IQRs was -0.498 (*p* = 0.002) for the affected eyes and -0.374 (0.016) for the fellow eyes.

ROC analysis revealed significant discriminative power for N2 peak time (AUC 73.97%; 95.00% CI: 65.06–82.88%), which was lower than that for the PVEP P1 peak time. The discrimination powers of the variability indices were as follows: SNR: 64.46%; 55.76–73.16%, IQ jitter: 55.00%; 45.52–64.49%, corresponding N: 60.42; 53.41–67.43%.

To explore whether the variability itself, even in the presence of normal peak time and amplitude, can detect optic neuritis with similar performance as increased peak times of the main peaks we performed an analysis based on the assumption that values for the pathologic variability would be greater than 95% of the variability in the fellow eyes. Using this method for PVEP, 11 eyes out of 33 were identified as pathologic in the corresponding N, 7 in the jitter IQR and 17 in the case of the SNR. In MVEP the rate was even lower, reflecting the fact that the variability of MVEP is usually greater even in healthy people: 4 eyes out of 33 were identified as pathologic in the corresponding N, 2 in the jitter IQR and 9 in the case of the SNR.

### Visual acuity (LogMAR)

The mean logMAR visual acuity of the fellow eyes was 0.126 (SD = 0.194) and that of the affected eyes was 0.521 (SD = 0.347). There was a significant difference in visual acuity between the fellow and affected eyes, which is in accordance with the diagnosis of acute unilateral optic neuritis (*p *< 0.001).

To test the possibility that increased variability in the affected eyes may be only an effect of poor fixation due to low visual acuity in the affected eye, we performed an additional analysis of the variability parameters of PVEP in a subgroup of twelve patients who had good VA even in the affected eyes (logMAR 0.00 – 0.30) which is not supposed to cause problems with fixation. Both the jitter IQR (*p* = 0.013) and corresponding N (*p* = 0. 007) revealed a significant difference between fellow and affected eyes in Wilcoxon paired test.

## Discussion

This study examined characteristics such as latency jitter (jitter IRQ, corresponding N) associated with single response variability. We also employed the signal‒to‒noise ratio (SNR) as the amplitude-related objective marker. We investigated whether these parameters could add some useful information, and possibly serve as an additional criterion in VEP assessment in the future. Such measurements might be beneficial in recordings with asymmetric or split peak, when reading of peak time and amplitude might not be reliable.

We found significant differences in the parameters of responses to the pattern-reversal stimulation (PVEP) and partially also the motion-onset stimulation (MVEP) between the eyes affected by unilateral optic neuritis (ON) and the fellow (healthy) eyes of the same subject. Following Kovarski et al. [9], we have supported that increased variability affects the amplitudes of the PVEP and MVEP.

The two stimulation types (PVEP, MVEP) were chosen because the PVEP predominantly activates the primary visual cortex via stimulation of the central parts of the retina whereas the MVEP is suggested to represent rather the magnocellular input of the dorsal stream [41, 42]. In some conditions, where the magnocellular pathway is predominantly affected, pathological findings can be observed only in MVEP, whereas PVEP is normal [20, 21]. Therefore, we were interested in whether MVEP variability also differs which was confirmed for some of the parameters.

Both central and peripheral fibers are affected by ON [17, 18, 22, 43]. Nevertheless, the central (macular–papillary) fibers of the optic nerve may be affected to a greater degree than the peripheral fibers are [44, 45]. This is reflected by abnormal electrophysiological findings by PVEP (which predominantly activates central fibers) [21, 46, 47] and less affected MVEP (which activates the central receptive fields to a lesser degree) [20]. Our results are in line with this concept because all five examined parameters were significantly different in the PVEP, and four of them were significantly different in the MVEP.

To evaluate whether the assessed markers can be used for diagnostic purposes, we performed an illustrative ROC analysis. The results demonstrated a significant ROC area under the curve for the SNR, jitter IQR, and peak time of the main peak. The peak time had superior discrimination power, but this was largely affected by the fact that increased peak times of the main peak were one of the inclusion criteria in our study.

We are aware that fellow (healthy) eyes with normal VEP peak times, may have subclinical affection because undiagnosed affection of the optic nerve in multiple sclerosis is not rare [48, 49]. We do not provide a comparison with a group of healthy controls in this proof of usability stage, and we concentrated on the interocular difference in variability of the affected and fellow eyes to lower the interindividual variability interference. We tried to minimize the risk of fellow eye affection, therefore, only patients whose fellow eye VEP recordings were normal were included. We excluded patients with a history of optic neuritis in both eyes because it is possible that some pathophysiologic changes (that may also increase the variability) may persist quite long after acute optic neuritis is resolved and that such an eye will not represent a suitable control. Nevertheless, we can never be absolutely sure that an asymptomatic eye is completely healthy.

Although we employed non-ISCEV filtering (bandpass from 0.3 to 45 Hz) and stimulus conditions for recording VEP, we applied these identically to both the affected and fellow eyes in each participant and the interocular comparisons should not be biased. However, different filter setup can limit the direct comparability of our results with studies using strictly standardized ISCEV protocol, and therefore generalizing these findings to different experimental or clinical contexts should be with caution.

We recognize that Woody’s method can, in principle, align random background activity rather than the true evoked response, as highlighted by Wastell [50]. In our study, we limited the maximum permissible latency shift (e.g., ± 30 ms). This approach reduces the likelihood of erroneously matching noise components (such as alpha activity) with the average waveform. Our study does not rely on the final aligned VEP waveforms for interpretative conclusions about amplitude, peak time, or morphology. Instead, our primary interest is in quantifying variability. If epochs have a low SNR, individual trial alignments will be more erratic, causing a larger jitter IQR and affecting how many trials meet the alignment criterion (corresponding N). Because the final shape of the aligned VEP is not our primary measure, any residual concern about “noise alignment” impacting the final average is much less critical. The limitation is that this approach cannot distinguish whether the variability seen across trials is due to true neural jitter or added noise. A limitation arises when the VEP amplitude is relatively small. The background noise becomes more significant relative to the signal, which can artificially inflate the measured variability (e.g., jitter estimates). Consequently, it can be difficult to discern whether heightened variability reflects underlying neural inconsistencies or is primarily due to noise contamination of a low-amplitude response. This confound underscores the need to interpret higher variability values cautiously when dealing with weak signals and use independently estimated noise to minimize this issue.

We acknowledge that we cannot identify and select which all sources of variability (not only pathologic conditions) influence particular VEP recordings; however, because most of them have approximately the same effect on the examination of both eyes, we feel unlikely that the increased variability is due to attention, recording or other factors. We suspect that eyes with ON may have higher VEP variability because their functions vary in time due to the influence of pathologic conditions as is likely the case also in other diagnoses [9, 12, 13].

To analyze the possible effect of poor fixation due to low visual acuity in the affected eyes, we performed an additional analysis of the variability parameters of PVEP in a subgroup of twelve patients who had good VA even in the affected eyes. Both the jitter IQR and the corresponding N revealed significant difference between fellow and affected eyes. Nevertheless, this problem would require deeper analysis.

For usage of these parameters, it is necessary to explore the variability in healthy controls, which is the subject of our ongoing study. Needed are also prospective studies including cases of ON with normal VEP findings to explore sensitivity and specificity of variability markers as in some of current results we detected increased fellow eye variability. The algorithmic basis of the variability detection might bring a diagnostic improvement in an untypical response or in other disorders where the standard VEP parameters do not provide such a good diagnostic outcome.

We do not overestimate the results of this study and currently, we cannot say that evaluation of the VEP variability parameters significantly increases the sensitivity of VEP examination in all their diagnostic applications. However, since their monitoring brings more information at no additional cost or less patient comfort, we believe that this approach should be tested whether to take it into account in diagnostic decisions.

## Conclusion

This study showed greater variability in single VEP trials in eyes affected by optic neuritis compared to fellow eyes. These findings may represent important pathophysiologic features. The study also suggested that the SNR, jitter IQR and corresponding N are algorithmic parameters that could reflect functional changes in pathological conditions. Adding (not replacing) them to traditional diagnostic markers (peak time and amplitude) could provide valuable information to VEP investigations.

## Data Availability

All data sets analyzed during the current study are available from the corresponding author upon reasonable request.

## Declarations

### Statement on the welfare of animals

This article does not contain any studies with animals performed by any of the authors.

## References

[1] Halliday AM, McDonald WI, Mushin J (1973) Visual evoked response in diagnosis of multiple sclerosis. Br Med J 4:661–664. 10.1136/bmj.4.5893.661 4758547 10.1136/bmj.4.5893.661 PMC1587677

[2] Marmoy OR, Viswanathan S (2021) Clinical electrophysiology of the optic nerve and retinal ganglion cells. Eye (Basingstoke). 10.1038/s41433-021-01614-x 10.1038/s41433-021-01614-x PMC8377055 34117382

[3] Dawson GD (1954) A summation technique for the detection of small evoked potentials. Electroencephalogr Clin Neurophysiol 6:65–84. 10.1016/0013-4694(54)90007-3 13141922 10.1016/0013-4694(54)90007-3

[4] Arieli A, Sterkin A, Grinvald A, Aertsen A (1996) Dynamics of ongoing activity: explanation of the large variability in evoked cortical responses. Science 273:1868–1871. 10.1126/science.273.5283.1868 8791593 10.1126/science.273.5283.1868

[5] Heinrich SP, Bach M (2001) Adaptation dynamics in pattern-reversal visual evoked potentials. Doc Ophthalmol 102:141–156. 10.1023/a:1017509717071 11518457 10.1023/a:1017509717071

[6] Mezer E, Bahir Y, Leibu R, Perlman I (2004) Effect of defocusing and of distracted attention upon recordings of the visual evoked potential. Doc Ophthalmol 109:229–238. 10.1007/s10633-004-8055-5 15957608 10.1007/s10633-004-8055-5

[7] Goris RLT, Movshon JA, Simoncelli EP (2014) Partitioning neuronal variability. Nat Neurosci 17:858–865. 10.1038/nn.3711 24777419 10.1038/nn.3711 PMC4135707

[8] Garrett DD, Kovacevic N, McIntosh AR, Grady CL (2011) The importance of being variable. J Neurosci 31:4496–4503. 10.1523/JNEUROSCI.5641-10.2011 21430150 10.1523/JNEUROSCI.5641-10.2011 PMC3104038

[9] Kovarski K, Malvy J, Khanna RK et al (2019) Reduced visual evoked potential amplitude in autism spectrum disorder, a variability effect? Transl Psychiatry. 10.1038/s41398-019-0672-6 31852886 10.1038/s41398-019-0672-6 PMC6920480

[10] MacDonald SWS, Nyberg L, Bäckman L (2006) Intra-individual variability in behavior: links to brain structure, neurotransmission and neuronal activity. Trends Neurosci 29:474–480. 10.1016/j.tins.2006.06.011 16820224 10.1016/j.tins.2006.06.011

[11] Arazi A, Censor N, Dinstein I (2017) Neural variability quenching predicts individual perceptual abilities. J Neurosci 37:97–109. 10.1523/JNEUROSCI.1671-16.2016 28053033 10.1523/JNEUROSCI.1671-16.2016 PMC6705669

[12] Castellanos FX, Sonuga-Barke EJS, Scheres A et al (2005) Varieties of attention-deficit/hyperactivity disorder-related intra-individual variability. Biol Psychiatry 57:1416–1423. 10.1016/j.biopsych.2004.12.005 15950016 10.1016/j.biopsych.2004.12.005 PMC1236991

[13] Milne E (2011) Increased intra-participant variability in children with autistic spectrum disorders: Evidence from single-trial analysis of evoked EEG. Front Psychol 2:. 10.3389/fpsyg.2011.00051 10.3389/fpsyg.2011.00051 PMC3110871 21716921

[14] Kelly JP, Darvas F, Weiss AH (2014) Waveform variance and latency jitter of the visual evoked potential in childhood. Doc Ophthalmol 128:1–12. 10.1007/s10633-013-9415-9 24146335 10.1007/s10633-013-9415-9

[15] Shors TJ, Ary JP, Eriksen KJ, Wright KW (1986) P100 amplitude variability of the pattern visual evoked potential. Electroencephalography and Clinical Neurophysiology/ Evoked Potentials 65:316–319. 10.1016/0168-5597(86)90010-9 2424744 10.1016/0168-5597(86)90010-9

[16] Morny EKA, Haldina J, Heinrich SP (2024) Simulating the effects of partial neural conduction delays in the visual evoked potential. Transl Vis Sci Technol. 10.1167/tvst.13.2.18 38386346 10.1167/tvst.13.2.18 PMC10896232

[17] Toosy AT, Mason DF, Miller DH (2014) Optic neuritis. Lancet Neurol 13:83–99. 10.1016/S1474-4422(13)70259-X 24331795 10.1016/S1474-4422(13)70259-X

[18] Kolappan M, Henderson APD, Jenkins TM et al (2009) Assessing structure and function of the afferent visual pathway in multiple sclerosis and associated optic neuritis. J Neurol 256:305–310. 10.1007/s00415-009-0123-z 19296047 10.1007/s00415-009-0123-z

[19] Camisa J, Bodis-Wollner I (1982) Stimulus parameters and visual evoked potential diagnosis. Ann N Y Acad Sci 388:645–647. 10.1111/j.1749-6632.1982.tb50828.x 6953896 10.1111/j.1749-6632.1982.tb50828.x

[20] Kuba M, Kubová Z, Kremláček J, Langrová J (2007) Motion-onset VEPs: characteristics, methods, and diagnostic use. Vis Res 47:189–202. 10.1016/j.visres.2006.09.020 17129593 10.1016/j.visres.2006.09.020

[21] Kubová Z, Kuba M, Spekreijse H, Blakemore C (1995) Contrast dependence of motion-onset and pattern-reversal evoked potentials. Vis Res 35:197–205. 10.1016/0042-6989(94)00138-C 7839616 10.1016/0042-6989(94)00138-c

[22] Ayadi N, Oertel FC, Asseyer S et al (2022) Impaired motion perception is associated with functional and structural visual pathway damage in multiple sclerosis and neuromyelitis optica spectrum disorders. Mult Scler J 28:757–767. 10.1177/13524585211032801 10.1177/13524585211032801 PMC8978464 34379018

[23] Hickman SJ, Petzold A (2022) Update on optic neuritis: an international view. Neuro-Ophthalmology 46:1–18. 10.1080/01658107.2021.1964541 35095131 10.1080/01658107.2021.1964541 PMC8794242

[24] Petzold A, Fraser CL, Abegg M et al (2022) Diagnosis and classification of optic neuritis. Lancet Neurol 21:1120–1134. 10.1016/S1474-4422(22)00200-9 36179757 10.1016/S1474-4422(22)00200-9

[25] Abel A, McClelland C, Lee MS (2019) Critical review: Typical and atypical optic neuritis. Surv Ophthalmol 64:770–779. 10.1016/j.survophthal.2019.06.001 31229520 10.1016/j.survophthal.2019.06.001

[26] Kumral D, Şansal F, Cesnaite E et al (2020) BOLD and EEG signal variability at rest differently relate to aging in the human brain. Neuroimage 207:116373. 10.1016/j.neuroimage.2019.116373 31759114 10.1016/j.neuroimage.2019.116373

[27] Brecelj J (2003) From immature to mature pattern ERG and VEP. Doc Ophthalmol 107:215–224. 10.1023/B:DOOP.0000005330.62543.9c 14711153 10.1023/b:doop.0000005330.62543.9c

[28] Odom JV, Bach M, Brigell M et al (2016) ISCEV standard for clinical visual evoked potentials: (2016 update). Doc Ophthalmol 133:1–9. 10.1007/s10633-016-9553-y 27443562 10.1007/s10633-016-9553-y

[29] Kremláček J, Kuba M, Chlubnová J, Kubová Z (2004) Effect of stimulus localisation on motion-onset VEP. Vis Res 44:2989–3000. 10.1016/j.visres.2004.07.002 15474572 10.1016/j.visres.2004.07.002

[30] Cant BR, Hume AL, Shaw NA (1978) Effects of luminence on the pattern visual evoked potential in multiple sclerosis. Electroencephalogr Clin Neurophysiol 45:496–504. 10.1016/0013-4694(78)90293-6 81751 10.1016/0013-4694(78)90293-6

[31] Acharya JN, Hani A, Cheek J et al (2016) American Clinical Neurophysiology Society guideline 2: guidelines for standard electrode position nomenclature. J Clin Neurophysiol 33:308–311. 10.1097/WNP.0000000000000316 27482794 10.1097/WNP.0000000000000316

[32] Göpfert E, Schlykowa L, Müller R (1988) Zur Topographie des Bewegungs-VEP am Menschen. Klin Neurophysiol 19:14–20. 10.1055/s-2008-1060848 3131104

[33] Kremláček J, Kuba M, Kubová Z et al (2007) Within-session reproducibility of motion-onset VEPs: effect of adaptation/habituation or fatigue on N2 peak amplitude and latency. Doc Ophthalmol 115:95–103. 10.1007/s10633-007-9063-z 17541662 10.1007/s10633-007-9063-z

[34] Woody CD (1967) Characterization of an adaptive filter for the analysis of variable latency neuroelectric signals. Med Biol Eng 5:539–554. 10.1007/BF02474247

[35] Schimmel H (1967) The (±) reference: accuracy of estimated mean components in average response studies. Science 157:92–94. 10.1126/science.157.3784.92 6026675 10.1126/science.157.3784.92

[36] The jamovi project (2022) jamovi. (Version 2.3). [Computer Software]. Retrieved from https://www.jamovi.org.

[37] Holm S (1979) A Simple Sequentially Rejective Multiple Test Procedure A Simple Sequentially Rejective Multiple Test Procedure. Source: Scandinavian Journal of Statistics 6:65–70

[38] R Core Team (2023) R Core Team (2023). _R: A Language and Environment for Statistical Computing. R Foundation for Statistical Computing, Vienna, Austria. <https://www.R-project.org/>. In: https://www.R-project.org

[39] Obuchowski NA (1997) Nonparametric Analysis of Clustered ROC Curve Data. Biometrics 53:. 10.2307/2533958 9192452

[40] Ying GS, Maguire MG, Glynn RJ, Rosner B (2022) Tutorial on biostatistics: receiver-operating characteristic (ROC) analysis for correlated eye data. Ophthalmic Epidemiol. 10.1080/09286586.2021.1921226 33977829 10.1080/09286586.2021.1921226 PMC8586066

[41] Shapley R (1982) Parallel pathways in the mammalian visual system. Ann N Y Acad Sci 388:11–20. 10.1111/j.1749-6632.1982.tb50781.x 6953863 10.1111/j.1749-6632.1982.tb50781.x

[42] Heinrich SP (2007) A primer on motion visual evoked potentials. Doc Ophthalmol 114:83–105. 10.1007/s10633-006-9043-8 17431818 10.1007/s10633-006-9043-8

[43] Raz N, Dotan S, Benoliel T et al (2011) Sustained motion perception deficit following optic neuritis: behavioral and cortical evidence. Neurology 76:2103–2111. 10.1212/WNL.0b013e31821f4602 21670440 10.1212/WNL.0b013e31821f4602

[44] Rinalduzzi S, Brusa A, Jones SJ (2001) Variation of visual evoked potential delay to stimulation of central, nasal, and temporal regions of the macula in optic neuritis. J Neurol Neurosurg Psychiatry 70:28–35. 10.1136/jnnp.70.1.28 11118244 10.1136/jnnp.70.1.28 PMC1763456

[45] Hickman SJ, Toosy AT, Jones SJ et al (2004) A serial MRI study following optic nerve mean area in acute optic neuritis. Brain 127:2498–2505. 10.1093/brain/awh284 15342363 10.1093/brain/awh284

[46] Blumhardt LD, Barrett G, Halliday AM, Kriss A (1978) The effect of experimental ‘scotomata’ on the ipsilateral and contralateral responses to pattern-reversal in one half-field. Electroencephalogr Clin Neurophysiol 45:376–392. 10.1016/0013-4694(78)90189-X 79476 10.1016/0013-4694(78)90189-x

[47] (2006) Origin of the Visual Evoked Potentials. In: Principles and Practice of Clinical Electrophysiology of Vision. The MIT Press, pp 207–234

[48] Klistorner A, Arvind H, Nguyen T et al (2009) Fellow eye changes in optic neuritis correlate with the risk of multiple sclerosis. Mult Scler 15:928–932. 10.1177/1352458509105228 19498018 10.1177/1352458509105228

[49] Green AJ, McQuaid S, Hauser SL et al (2010) Ocular pathology in multiple sclerosis: retinal atrophy and inflammation irrespective of disease duration. Brain 133:1591–1601. 10.1093/brain/awq080 20410146 10.1093/brain/awq080 PMC2877904

[50] Wastell DG (1977) Statistical detection of individual evoked responses: an evaluation of Woody’s adaptive filter. Electroencephalogr Clin Neurophysiol 42:835–839. 10.1016/0013-4694(77)90238-3 67936 10.1016/0013-4694(77)90238-3

